# The differential diagnostic value of a battery of oculomotor evaluation in Parkinson's Disease and Multiple System Atrophy

**DOI:** 10.1002/brb3.2184

**Published:** 2021-05-30

**Authors:** Hong Zhou, Xia Wang, Di Ma, Yanyan Jiang, Fan Li, Yunchuang Sun, Jing Chen, Wei Sun, Elmar H Pinkhardt, Bernhard Landwehrmeyer, Albert Ludolph, Lin Zhang, Guiping Zhao, Zhaoxia Wang

**Affiliations:** ^1^ Department of Neurology Peking University First Hospital Beijing China; ^2^ Department of Neurology Ulm University Ulm Germany; ^3^ Department of Neurology UC Davis School of Medicine Sacramento CA USA

**Keywords:** Eye Movement Disorders, multiple system atrophy, Parkinson's disease, Smooth Pursuit Deficiency

## Abstract

**Introduction:**

Clinical diagnosis of Parkinsonism is still challenging, and the diagnostic biomarkers of Multiple System Atrophy (MSA) are scarce. This study aimed to investigate the diagnostic value of the combined eye movement tests in patients with Parkinson's disease (PD) and those with MSA.

**Methods:**

We enrolled 96 PD patients, 33 MSA patients (18 with MSA‐P and 15 with MSA‐C), and 40 healthy controls who had their horizontal ocular movements measured. The multiple‐step pattern of memory‐guided saccade (MGS), the hypometria/hypermetria of the reflexive saccade, the abnormal saccade in smooth pursuit movement (SPM), gaze‐evoked nystagmus, and square‐wave jerks in gaze‐holding test were qualitatively analyzed. The reflexive saccadic parameters and gain of SPM were also quantitatively analyzed.

**Results:**

The MGS test showed that patients with either diagnosis had a significantly higher incidence of multiple‐step pattern compared with controls (68.6%, 65.2%, and versus. 2.5%, *p* < .05, in PD, MSA, versus. controls, respectively). The reflexive saccade test showed that MSA patients showing a prominent higher incidence of the abnormal saccade (63.6%, both hypometria and hypermetria) than that of PD patients and controls (33.3%, 7.5%, respectively, hypometria) (*p* < .05). The SPM test showed PD patients had mildly decreased gain among whom 28.1% presenting “saccade intrusions”; and that MSA patients had the significant decreased gain with 51.5% presenting “catch‐up saccades”(*p* < .05). Only MSA patients showed gaze‐evoked nystagmus (24.2%), square‐wave jerks (6.1%) in gaze‐holding test (*p* < .05).

**Conclusions:**

A panel of eye movements tests may help to differentiate PD from MSA. The combined presence of hypometria and hypermetria in saccadic eye movement, the impaired gain of smooth pursuit movement with “catch‐up saccades,” gaze‐evoked nystagmus, square‐wave jerks in gaze‐holding test, and multiple‐step pattern in MGS may provide clues to the diagnosis of MSA.

## Graphical abstract

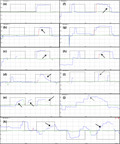

## 1 INTRODUCTION

Clinical diagnosis of PD is supported by a combination of the cardinal signs of resting tremor, bradykinesia, rigidity, and loss of postural reflexes (Postuma et al., 2015). In contrast, the diagnosis and management of patients with multiple system atrophy (MSA), especially within the first few years of disease, can be clinically challenging. MSA is characterized by prominent parkinsonism with additional features including cerebellar ataxia, early autonomic dysfunction, and pyramidal tract signs. Currently, the common way to differentiate MSA from PD is to wait and see how the disease progresses, with MSA advancing more rapidly than PD. In addition, the presence of cerebellar symptoms and signs supports the diagnosis of MSA. Some laboratory examinations might provide differential diagnostic clues for PD and MSA, such as imaging techniques, genetic testing, and measurement of biological markers. In addition, the tests of sleep disorders, neurobehavioral symptoms, olfactory dysfunction, and autonomic nervous system are also helpful for the diagnosis of the disease (Waragai et al., 2013). However, the diagnostic biomarkers of MSA are scarce, especially in its early stages (Fanciulli et al., 2019).

Recently, eye movement assessment, with the advantage of being noninvasive, offers insight into the underlying neural mechanisms in some neurodegenerative disorders and is considered as a potential diagnostic biomarker for parkinsonism (Anderson et al., 2008; Anderson & MacAskill, 2013; Gorges et al., 2014; Pinkhardt & Kassubek, 2011; Termsarasab et al., 2015; White et al., 1983). Particularly, the finding of cerebellar eye movement abnormality, such as positioning downbeat nystagmus, gaze‐evoked nystagmus, skew deviation, and macro square‐wave jerks, strongly points to MSA over PD, which can be detected by bedside clinical examination, even in the early disease course (Anderson et al., 2008). Video‐oculography (VOG) could further facilitate recording of precise and accurate eye movements and quantify eye movement recordings under computer‐controlled paradigms, making it a more sensitive examination for detecting subtle ocular motor pathology than bedside clinical examination. Saccadic pathology, such as mild saccadic hypometria, multiple saccadic steps patterns in reflexive saccade, and slightly decreased gain in SPM, has been found in PD patients (Anderson et al., 2008; Anderson & MacAskill, 2013; Gorges et al., 2014; Termsarasab et al., 2015; White et al., 1983). In MSA patients, on the other hand, mild to moderate saccadic hypometria and/or hypermetria, as well as mild to moderately reduced gain in SPM, positioning downbeat nystagmus, gaze‐evoked nystagmus as well as macro square‐wave jerks have been reported in recent studies (Anderson et al., 2008; Anderson & MacAskill, 2013; Pinkhardt & Kassubek, 2011; Pinkhardt et al., 2009). Kimmig et al and Blekher et al. reported a multiple‐step pattern in memory‐guided saccade (MGS) as a distinctive phenomenon in PD patients compared with normal controls (Blekher et al., 2009; Kimmig et al., 2002). However, whether multiple‐step pattern is specific for PD remains to be elucidated. In addition to saccade tests, smooth pursuit tests had also been examined in PD and MSA patients, with PD patients showing repeatedly interspersed “anticipatory saccades” or superimposed by “saccadic intrusions”(Gorges et al., 2013; Gorges et al., 2014; Pinkhardt et al., 2008; Pinkhardt & Kassubek, 2011); whereas MSA patients presenting “multiple small steps” was also described in the saccade, and SPM was described as necessitated “catch‐up saccades”(Anderson et al., 2008; Pinkhardt et al., 2009). Besides, patients with predominant cerebellar ataxia (MSA‐C) usually present with cerebellar eye signs such as gaze‐evoked, positional downbeat nystagmus, and rebound nystagmus, and MSA‐P have increased square‐wave jerks (Anderson & MacAskill, 2013).Up to now, most previous studies only detected reflexive saccadic and/or smooth pursuit tests, gaze‐holding test, while the studies demonstrating whether a battery of different eye movement tests(including MGS) could improve the diagnostic efficiency for discriminating PD form MSA are limited (Anderson et al., 2008; Pinkhardt et al., 2009). Herein, we aimed to investigate the potential value of the combination of eye movement tests between patients with PD and MSA.

## 2 MATERIALS AND METHODS

### 2.1 Subjects and clinical assessments

All participants of this retrospective study were enrolled from the first hospital of Peking University Movement Disorders Clinic between 2018.06 and 2019.12. The sample included (1) patients with idiopathic PD (*n* = 96); (2) patients with MSA (*n* = 33, 18 with MSA‐P and 15 with MSA‐C); and (3) healthy individuals (healthy controls, *n* = 40). Three movement disorder neurologists (ZXW, JC, WS) made the diagnoses according to the criteria established by Movement Disorders Society and the consensus statement on the diagnosis of MSA (Gilman et al., 2008; Postuma et al., 2015). We collected clinical data of all participants, including their age of onset, disease duration, onset symptoms, and complications. PD patients were assessed using the Unified Parkinson Disease Rating Scale (Movement Disorder Society, 2003). None of the healthy controls were on medication of movement disorder‐related diseases, or presented with any other symptoms and signs of nervous system diseases, had substance abuse or dependence, or any psychiatric disorders according to DSM‐V criteria, vestibular system disease, or cataract. The Mini‐Mental State Examination (MMSE) was used to assess cognitive function. Patients with severe dementia who cannot cooperate to complete eye movement tests or poor quality of eye movement data were excluded. This study was approved by the Ethical Committee of Peking University First Hospital. All study participants were provided with and signed written informed consent for participation.

The demographic and clinical characteristics for the PD subjects, MSA subjects, and the controls were shown in Table 1. Most of the PD participants were mild to moderately affected with a mean H&Y of 1.72 ± 0.78 (range 1–4). None of the participants had an additional, concurrent neurological illness, severe mental disorders (i.e., bipolar, schizophrenia), or a history of alcohol or drug abuse (Association, 2013). All participants had normal or corrected visual acuity and did not report significant eye‐related complaints. Ninety‐five percent of the PD patients were taking medication to treat their neurological disorder; they were instructed to continue taking their medications. MSA patients were under appropriate treatment if they were needed.

**TABLE 1:** Demographic and clinical characteristics of the three study groups

Demographic/Clinical data	PD (*n* = 96)	MSA (*n* = 33)	HC (*n* = 40)	F/χ^2^,*p*
Age, year	62.46 ± 13.31	59.31 ± 8.94	60.78 ± 15.78	0.53, *p* = .58
M/F	52/44	15/18	17/23	1.554, *p* = .46
Disease duration, months	62.03 ± 6.84	24 (6,36)	‐	3.10, *p* = .08
Hoehn & Yahr stage	(1 ~ 1.5) 40	‐	‐	
	(2 ~ 2.5) 49			
	(3 ~ 4) 7			
UPDRS (III)	17 (9,29)			
MMSE	26.9 ± 3.43 (20,30)	23.50 ± 4.20 (18,30)^*^	28.5 ± 2.34	3.30, ^*^ *p* < .001

Abbreviations: F, female; HC, healthy controls; M, male; MMSE, Minimum Mental State Examination; MSA, multiple system atrophy; PD, Parkinson's disease; UPDRS, the Unified Parkinson Disease Rating Scale.

^*^ *p* .05

### 2.2 Record

A binocular EyeLink system (Bao Runtong Research Ltd. China) was used for video‐oculographic eye movement recordings. The measurement took place in our dedicated oculomotor laboratory. All participants were comfortably seated facing the center of a black hemicylindrical screen (the eyes‐to‐screen distance was approximately 120 cm). Subjects were required to keep their heads still while move eyes according to the instructions. All tasks were tested in a dark room. The targets on the screen are red LED lights. Ocular motor testing was completed during a 30‐min session.

#### 2.2.1 Testing procedure

We performed four eye movement tasks in a fixed order. The equipment was calibrated at the beginning of the tasks. Before each task, the examiner instructed the participant verbally and completed a practice demonstration to ensure that the participant understood the oral instructions correctly.

*MGS*: The participant was instructed to fix their gaze on the central spot for 2 s, then a horizontal peripheral stimulus (“target”) was presented for a period of 3 s at the same time. The participant was required to keep the fixation on the central spot until the target was switched off, and the participant was required to keep fixating on the central spot, which would be switched off after a 2 s' delay. When the central spot disappeared, the participant was required to saccade immediately toward the remembered location of the target and fixate on the memorized location for 3 s. When the central spot reappeared, the participant was required to fix their gaze on the central spot for the next test (Figure S1). The targets were located on the screen for ±15°, ±20°, and ±25° on each location tested repeated for five times.

*Reflexive saccade*: The participant was required to fixate on the central spot (0°). The primary target extinguished simultaneously with the illumination of a peripheral LED. Timing (1.0–1.5 s) and position of the horizontal LED (2 times of ±5°, ±10°, ±15°, ±20°, ±25°, and ±30°) appeared randomly on the screen. The participant was instructed to visually track the target light as rapidly as possible so that each target step proceeded with the previous step.

*SPM*: The participant was asked to fixate at a sinusoidally moving target smoothly. The target moved in the horizontal direction (amplitude ± 15°) at a frequency of 0.2 Hz (6 cycles = 30 s).

*Gaze‐holding test*: The participant was asked to fixate at a target in a central position and then in eccentricities of ±15° horizontally, 10 s in each position.

#### 2.2.2 Measurements of eye movements

##### Quantitative measures

*Reflexive saccades*: After the participant completed the testing procedure, an interactive computerized analysis was carried out to quantify primary saccade measures for reflexive saccade task. The latency (the interval between target presentation and the start of the saccade), peak velocity, and accuracy/gain (the ratio of saccade amplitude to target amplitude) were quantified for each saccade angle (Blekher et al., 2009; R. John Leigh, 2015).

*SPM*: The gain of SPM (eye velocity/target velocity) was calculated separately by computer for the left and the right directions, which was the average of respective half‐cycles' value of each direction (Figure S2) (R. John Leigh, 2015). A gain of 1.0 indicates that the actual eye movement is perfectly aligned with the moving target.

##### Qualitative measures

*hypometria/hypermetria and saccade intrusion* in reflexive saccade: defined as the degree of accuracy decreased or increased by 10% of amplitude (Gorges et al., 2013; R. John Leigh, 2015).

*multiple‐step pattern* in MGS: the incidence of participants with at least one gaze shift consisting of 3 or more saccades made in the same direction, and the curve turning angle of eye movement recording in memory saccade was 90° (classical “multiple‐step pattern”) was computed (Blekher et al., 2009). Unwanted saccades were defined as a reflexive saccade in MGS that is not needed or an advanced saccade before instruction was observed.

*Anticipatory saccade, catch up saccade, and saccade intrusion* in SPM. Anticipatory saccade: anticipate the future target position in SPM. Catch up saccade: saccades typically correct for the defective SPM. Saccade intrusion: Inappropriate saccadic movements that move the line of sight away from the object of regard: almost standard SPM curve superimposed saccades. (Gorges et al., 2013; Gorges et al., 2014; R. John Leigh, 2015; Pinkhardt et al., 2009).

*Gaze‐evoked nystagmus, square‐wave jerks, macro square‐wave jerks in gaze‐holding test*: Gaze‐evoked nystagmus is present when gaze is directed eccentrically, to right or left. Square‐wave jerks: pairs of small horizontal saccades (typically <2°) that take the eye away from the target and then return it within 200 ms; often occur in a series. Macro square‐wave jerks: large (5–15°) saccadic intrusions that take the eye away from the target and return it within 70–150 ms. (R. John Leigh, 2015).

### 2.3 Statistical analysis

Latency, accuracy, and velocity of reflexive saccade as well as gain of SPM were summarized by mean (*SD*) or median [interquartile range (IQR)] for skewed variables (e.g., duration of patients). Analysis of variance (ANOVA) was used to compare mean levels of the data mentioned above, and every two groups’ parameters were compared if there were significant differences. The chi‐square test was used for assessing the difference in the distribution of a categorical variable by different diseases (e.g., rates of multiple‐step pattern, abnormal saccade in SPM). The diagnostic sensitivity and specificity of combined eye movement from PD to MSA were calculated. A *p*‐value < .05 was considered statistically significant.

## 3 RESULTS

### 3.1 Memory‐guided saccade (MGS)

**FIGURE 1:**
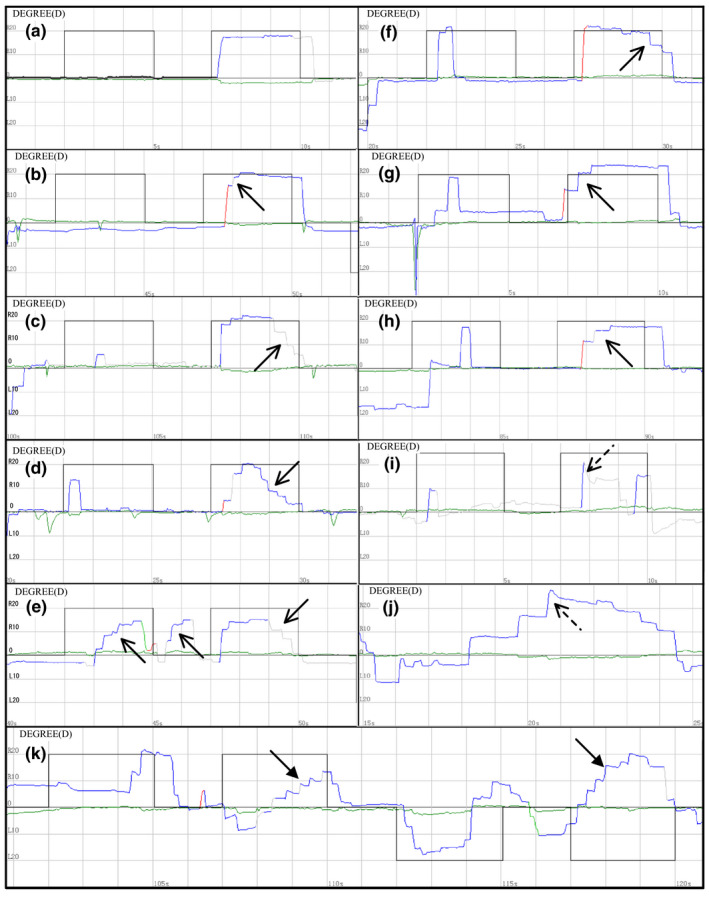
Multiple‐step pattern in memory‐guided saccade. (a) A 64‐year‐old male PD patient (H&Y 1) showed a completely normal performance in MGS. The target located horizontally right 20°. Blackline is the target reference curve, and the color line is the actual eye movement. (b) A 38‐year‐old male healthy control with multiple‐step pattern. (c) A 64‐year‐old female PD patient (H&Y 1) showed multiple‐step pattern when saccade to the central target at the end of the task. (d) A 61‐year‐old female PD patient (H&Y 1) showed multiple‐step pattern not only when saccade to the memory target, but also when saccade to the central target at the end of the task. (e) A 54‐year‐old male PD patient (H&Y 1) showed multiple‐step pattern in MGS and the unwanted saccades. (f) A 54‐year‐old male PD patient (H&Y 1) showed multiple‐step pattern when saccade to the central target at the end of the task. (g) A 64‐year‐old male PD patient (H&Y 2) showed multiple‐step pattern when saccade to the memory target. (h) A 51‐year‐old female MSA patient showed the typical multiple‐step pattern in MGS. (i) A 59‐year‐old female MSA patient showed hypermetria in a task. (j) A 47‐year‐old female MSA showed hypermetria and multiple‐step pattern. (k) A 64‐year‐old female MSA patient showed atypical multiple‐step pattern with the angle of multi‐step less than 90° (bold arrow). And a saccade error could be seen at the end of the mission, saccade to the opposite memorized direction. Multiple‐step pattern: solid arrow, hypermetria: dotted arrow

The incidence of multiple‐step pattern was 68.6% (66/96) in PD, 65.2% (15/23 in MSA, 14 with MSA‐P; Table 2), and 2.5% (1/40) in healthy controls (Figure 1). There was a significant difference both between PD versus. controls and between MSA versus. controls (χ^2^ = 51.62, *p* = .001). However, there was no significant difference between PD and MSA (χ^2^ = 0.11, *p* = .74). Both PD and MSA patients could finish the MGS tests. In a single MGS trial of the PD patients, multiple‐step pattern occurred not only when saccade to the memorized target, but also when saccade to the central target at the end of the task. In the MSA patients, the typical multiple‐step pattern could also be present in MGS tests. Additionally, in 17.4% (4/23) MSA patients, MGS could be accompanied by hypermetria (3 with MSA‐C, 1 MSA‐P patients with clinical cerebellar features), or the curve turning angle of multiple‐step pattern less than 90°(10/23, 4 with MSA‐C), which presented as atypical multiple‐step pattern, mimicking nystagmus.

**TABLE 2:** Eye movement recordings of all participants

Tasks		PD	MSA	HC	F/χ^2^,*p*
MGS (multiple‐step pattern)	(*N*/%)	66 (68.6%) (*n* = 96)	15 (65.2%) (*n* = 23)	1 (2.5%) (*n* = 40)	318, ^*^ *p *< .001
Gaze‐holding test	gaze‐evoked nystagmus (*N*/%)	0	8 (24.2%) (*n* = 33)	0	34.6, ^*^ *p *< .001
	square‐wave jerks (*N*/%)	0	2 (6.1%)	0	8.34, ^*^ *p* = .015
Reflexive saccade	Abnormal (*N*/%)	32 (33.3%) (*n* = 96)	21 (63.6%) (*n* = 33)	3 (7.5%) (*n* = 40)	338, ^*^ *p *< .001
	Hypometria (*N*/%)	32 (33.3%)	16 (48.5%)	3 (7.5%)	
	Hypermetria (*N*/%)	0	1 (3.0%)	0	
	Hypermetria and hypometria (*N*/%)	0	4 (12.1%)	0	
Saccade intrusions in reflexive saccade		17 (17.7%)	8 (24.2%)	3 (7.5%)	3.88, ^*^ *p *= .015
SPM	Abnormal (*N*/%)	42 (43.8%) (*n* = 96)	20 (60.6%) (*n* = 33)	4 (10.0%) (*n* = 40)	338, ^*^ *p *< .001
	Intrusions (*N*/%)	22 (22.9%)	2 (6.0%)	2 (5.0%)	
	Anticipatory saccade (*N*/%)	10 (10.8%)	1 (3.0%)	0	
	Catch‐up saccade (*N*/%)	5 (5.2%)	13 (39.4%)	0 (0%)	
	Intrusions+Anticipatory saccade	4 (4.2%)	0	2 (5.0%)	
	Intrusions+Catch‐up saccade	1 (1.0%)	1 (3.3%)	0	
	Intrusions+Anticipatory saccade+Catch‐up saccade	0	3 (9.1%)	0	
	Gain (mean ± *SD*) toward Left	0.76 ± 0.12	0.65 ± 0.15	0.79 ± 0.10	8.51, ^*^ *p *< .001
	Gain (mean ± *SD*) toward Right	0.73 ± 0.12	0.61 ± 0.15	0.76 ± 0.10	11.16, ^*^ *p *< .001

Abbreviations: HC, healthy controls; MGS, memory‐guided saccade; MSA, multiple system atrophy; PD, Parkinson's disease; *SD*, standard deviation; SPM, Smooth pursuit movement.

^*^ *p* .05

### 3.2 Reflexive saccade

**FIGURE 2:**
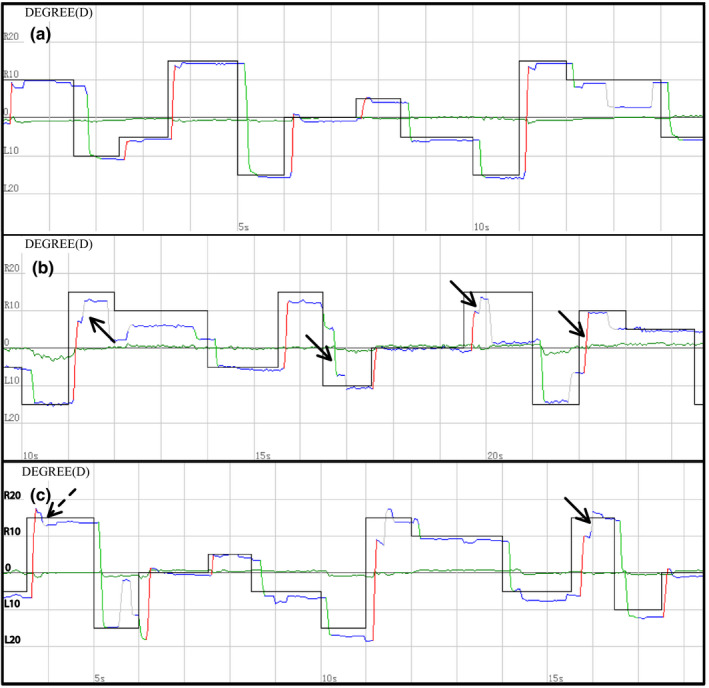
Reflexive saccade of all participants. (a) A 31‐year‐old male healthy control showed perfect performance. Blackline is the target reference line, and the color line is the actual eye movement. (b) A 63‐year‐old female PD patient (H&Y1) showed hypometria. (c) A 56‐year‐old female MSA patient showed both hypometria and hypermetria. hypometria: solid arrow, hypermetria: dotted arrow

Reflexive saccade pathology occurred in 32 PD (32/96, 33.3%) and 21 MSA (21/33, 63.6%) patients, which was significant higher than in healthy controls (3/40, 7.5%;χ^2^ = 9.86 *p* = .002, χ^2^ = 25.82，*p* < .001, respectively; Table 2). Hypometria could be found in PD patients (33.3%) and MSA patients (60.6%), but hypermetria could be found only in MSA‐C patients (15.2%) (Figure 2). The latency, accuracy, and velocity of reflexive saccade in each angle were compared among different groups. The shortening latency on the location of +10°, −15°, ±20°, ±25°, and ±30° in PD patients showed a significant difference compared with healthy control (*p* < .05; Table 3). Saccade intrusions occurred in 17 PD (17/96, 17.7%) and 8 MSA (8/33, 24.2%) patients, which was higher than in healthy controls (3/40, 7.5%) (*p* > .05; Table 2).

**TABLE 3:** The parameters of reflexive saccade in PD, MSA and HC

		−5°	−10°	−15°	−20°	−25°	−30°	+5°	+10°	+15°	+20°	+25°	+30°
Latency ms, (mean ± *SD*)	HC	234.50 ± 100.36	257.50 ± 66.44	225.00 ± 55.05	267.50 ± 83.60,	247.00 ± 72.68	241.50 ± 71.38	200.52 ± 48.88	216.00 ± 65.19	232.50 ± 71.56	223.00 ± 58.09	272.00 ± 68.66	259.00 ± 52.42
	PD	187.69 ± 139.07	226.67 ± 93.59	173.55 ± 74.49^a^,^e^	229.46 ± 86.86^a^,^d^	213.33 ± 90.76^a^,^d^	195.69 ± 51.06^a^,^e^	171.08 ± 105.17	177.20 ± 95.94^a^,^d^	199.35 ± 112.81	195.27 ± 68.42^a^,^d^	227.09 ± 95.40^a^,^d^	214.19 ± 71.17^a^,^d^
	MSA	218.00 ± 135.94	222.00 ± 51.87	188.00 ± 50.01^b^,^d^	211.00 ± 59.28^b^,^d^	261.00 ± 94.36^c^,^d^	225.00 ± 56.14^c^,^d^	182.22 ± 41.09	213.00 ± 151.52	200.00 ± 43.53^c^,^d^	243.00 ± 69.37	257.00 ± 103.47	242.00 ± 82.05
Accuracy %, (mean ± *SD*)	HC	96.97 ± 16.18	91.46 ± 11.74	88.75 ± 12.65	87.53 ± 9.94	88.17 ± 12.91	80.98 ± 14.03	95.40 ± 17.54	91.73 ± 12.50	89.10 ± 11.32	88.35 ± 12.48	88.40 ± 15.09	85.28 ± 14.25
	PD	100.64 ± 29.17	90.57 ± 15.98	80.14 ± 17.67	85.67 ± 17.60^a^,^d^	81.61 ± 17.78	80.32 ± 17.22	98.78 ± 24.84	89.16 ± 20.32	86.13 ± 14.42	84.82 ± 15.99	82.46 ± 17.50	80.91 ± 17.02
	MSA	98.89 ± 21.91	85.55 ± 12.86	79.10 ± 20.07^b^,^d^	73.95 ± 18.26^b^,^d^	83.75 ± 19.14	79.15 ± 15.41	92.23 ± 21.26	84.95 ± 25.07	92.65 ± 15.35	89.35 ± 13.81	78.85 ± 22.66	81.05 ± 14.94
Velocity °/s, (mean ± *SD*)	HC	118.07 ± 17.15	179.33 ± 31.79	247.45 ± 44.19	285.20 ± 53.29	333.87 ± 70.36	333.95 ± 64.77	122.47 ± 25.43	197.08 ± 32.70	254.62 ± 45.53	292.75 ± 49.45	331.28 ± 64.39	356.85 ± 61.16
	PD	118.57 ± 22.18	186.70 ± 37.40^c^,^d^	228.20 ± 60.07	286.53 ± 63.63	307.84 ± 61.62^a^,^d^	341.11 ± 87.30	118.72 ± 24.78	188.16 ± 39.65	250.81 ± 61.09	294.30 ± 61.40	328.98 ± 77.43	346.11 ± 79.73
	MSA	117.50 ± 25.5	165.10 ± 26.42	232.70 ± 59.58	274.50 ± 68.65	343.95 ± 84.60^b^,^d^	357.25 ± 67.80	112.16 ± 34.68	187.20 ± 31.39	265.50 ± 57.01	297.65 ± 54.63	312.95 ± 83.59	344.55 ± 71.24

Note

‐ saccade direction: left, + saccade direction: right.

Abbreviations: HC, healthy controls; MSA, multiple system atrophy; PD, Parkinson's disease; *SD*, standard deviation.

^a^ PD compared with HC.

^b^ MSA compared with HC.

^c^ PD compared with MSA.

^d^ *p *< .05.

^e^ *p *< .001.

### 3.3 SPM

**FIGURE 3:**
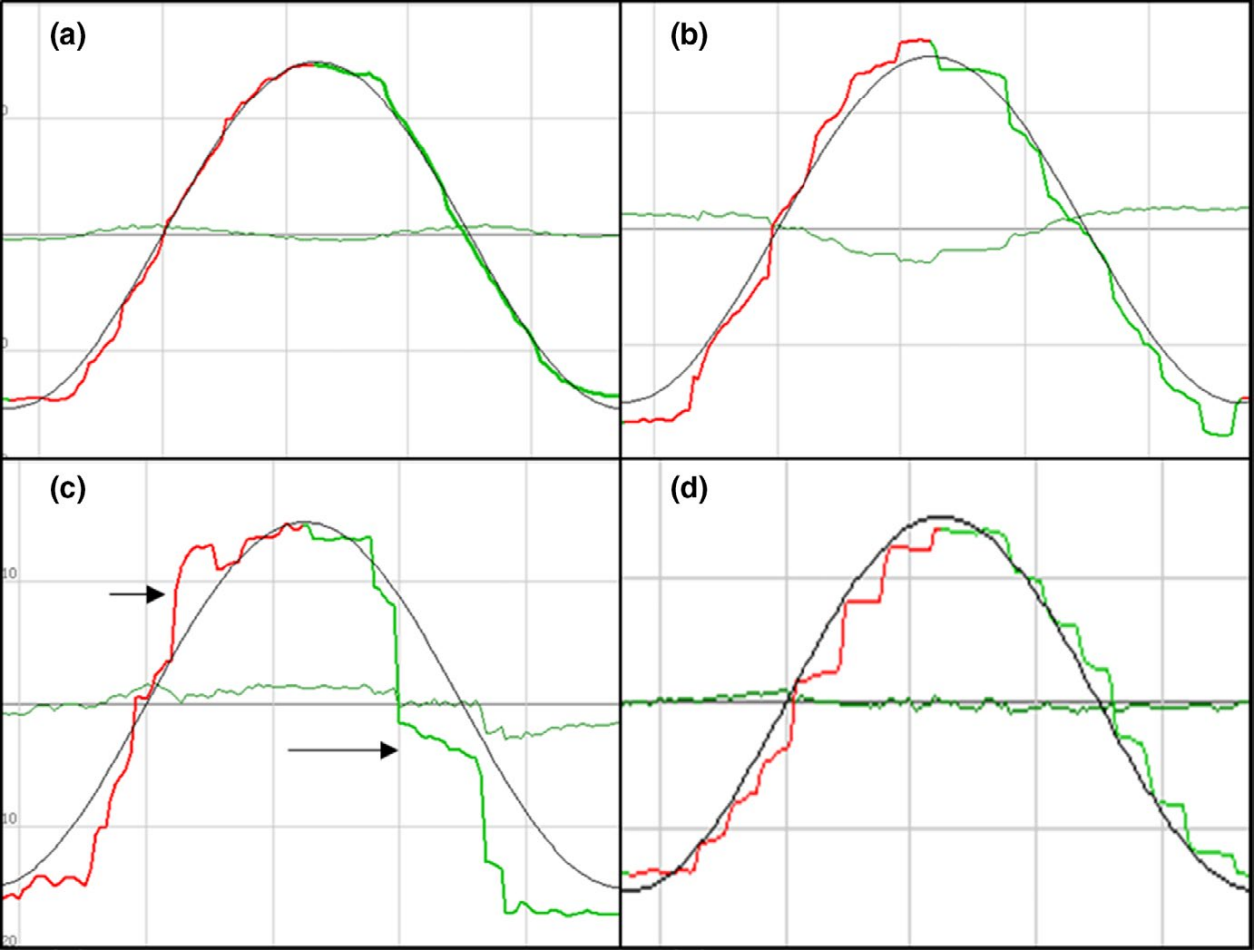
The recordings illustrating the participants' SPM during tracking of a sinusoidal horizontal target movement. Blackline is the target reference line, and the color line is the actual eye movement. (a) a 25‐year‐old female healthy control showed perfect performance. (b) a 70‐year‐old female PD patient (H&Y 3) with the disease duration of 9 years, “switches” between time‐points of “anticipatory saccades” with slightly reduced gain in SPM. (c) a 74‐year‐old female PD patient (H&Y 2.5) with the disease duration of 2 years showed exclusively “anticipatory saccades” (long arrow) to track the target and “saccade intrusions” (short arrow). (d) a 47‐year‐old female MSA patient with the disease duration of 3 years showed a regular sequence of “catch‐up saccades”

Compared to healthy controls (eye movements toward the left and the right directions: 0.79 ± 0.10 and 0.76 ± 0.10, respectively), SPM gain were slightly decreased in PD patients (left and right: 0.76 ± 0.12 and 0.73 ± 0.12, respectively), SPM gain in MSA patients were reduced (left and right: 0.65 ± 0.11 and 0.61 ± 0.15, respectively) (Table 2). The gain of SPM in MSA patients was significantly lower than PD patients (toward left and right, *t* = −4.64, *p* < .001 and *t* = −4.64, *p* < .001, respectively) and healthy controls (toward left and right, *t* = −5.69, *p* < .001 and *t* = −5.10, *p* < .001, respectively), but no significant difference was seen between PD patients and healthy controls (toward left and right, *t* = −1.39, *p* = .17 left = right). There were 43.8% of PD patients (42/96), 60.6% of MSA patients (20/33), and 10.0% of healthy controls (4/10) showed abnormal saccades in SPM. The presence of “saccade intrusions” was found in 28.1% of PD patients, 18.2% of MSA patients (PD versus. MSA, χ^2^ = 1.27, *p* = .35), and 10.0% of healthy controls (χ^2^ = 5.27, *p* = .03). The presence of “catch‐up saccades” occurred in 51.5% of MSA patients, in 6.3% of PD patients (MSA versus. PD, χ^2^ = 34.34, *p* < .001). No healthy control showed the “catch‐up saccades.” Anticipatory saccade could be found in 14.6% of PD patients, 12.1% of MSA patients, and 5% of healthy controls (χ^2^ = 0, *p* = 1.0) (Table 2, Figure 3, Figure S3, Figure S4).

### 3.4 Gaze‐holding test

Compared with PD patients and healthy controls, only MSA patients had abnormal gaze‐evoked nystagmus (8/33, 24.2%) and square‐wave jerks (2/s, 2/33, 6.1%) (*p* < .05), no macro square‐wave jerks were found in all participants (Table 2).

### 3.5 Discrimination between PD and MSA

Compared to PD patients, MSA patients showed hypometria and hypermetria, the decreased gain of smooth pursuit movement with “catch‐up saccades,” gaze‐evoked nystagmus, square‐wave jerks in gaze‐holding test, and multiple‐step pattern in MGS. The sensitivity of abnormal signs mentioned above in diagnostic performance of MSA versus PD was 72.7% (24/33), and the specificity was 98.9% (95/96).

## 4 DISCUSSION

Due to the similarity and the considerable overlap of oculomotor symptoms among Parkinsonian syndromes, most authorities in the field believe that the diagnostic advantage of eye movements is rather limited in differentiating PD from MSA only by oculomotor testing, especially in their early stages (Anderson et al., 2008; Anderson & MacAskill, 2013; Pinkhardt et al., 2008). However, multiple‐step pattern was considered as a potential biomarker in PD according to a recent study (Blekher et al., 2009). The study by Pinkhardt et al. also suggested that the differences in SPM between PD and MSA were significant enough to warrant their use as ancillary diagnostic criteria for the distinction between these two disorders, as cerebellary driven catch up saccades are a sole feature of MSA in contrast to PD, even if seen subclinically by VOG (Pinkhardt et al., 2009). Our data showed that multiple‐step pattern of MGS could occur not only in PD patients, but also in a high proportion of MSA patients. Quantitative analyses of reflexive saccade parameters alone were not so distinctive, however. Nevertheless, a battery of cerebellar oculomotor characteristics consisting of hypermetria, smooth pursuit movement with “catch‐up saccades,” gaze‐evoked nystagmus, square‐wave jerks, and multiple‐step pattern in MGS could provide potential clues for differentiating MSA from PD.

A good detailed ocular motor bedside clinical examination is very useful for detecting ocular abnormalities. Further, VOG could make eye movement recording a more sensitive examination for detecting subtle ocular motor pathology than bedside clinical examination. Blekher et al. reported that multiple‐step pattern of MGS tasks demonstrated good sensitivity (87%) and excellent specificity (96%) in the ability to discriminate PD patients from controls (Blekher et al., 2009). However, the incidence of multiple‐step pattern in PD in our study was not so high. Furthermore, our study showed an almost equally high incidence of multiple‐step pattern in PD (68.6%) and MSA patients (65.2%), indicating that multiple‐step pattern is not unique to PD. In PD, the occurrence of multiple‐step pattern was attributed to the dysfunction of the basal ganglia dopaminergic network, subthalamic nucleus (STN), or functional connectivity of the brain (Gorges et al., 2013; Harting & Updyke, 2006; Rivaud‐Péchoux et al., 2000). We inferred that in MSA, similar brain pathology of PD might exist, resulting in the typical multiple‐step pattern of MGS, since Halliday et al. had reported that neuronal loss and glial cytoplasmic inclusions might happen in the basal ganglia as part of the pathology of MSA (Halliday et al., 2011). Yet, further analysis revealed that MSA patients also showed atypical features compared with that in PD patients. We speculate that it is probably the result of the interaction of impaired basal ganglia and cerebellum, since previous studies showed the lesions of cerebellar input and output pathways could also result in multiple short‐latency hypometric saccades to achieve the visual target in SCA patients (Kheradmand & Zee, 2011; Rucker et al., 2006). Additionally, the atypical multiple‐step pattern in MSA maybe resulted from the mismatch of saccade control neurons and the impaired output information of the neural integrator associated with cerebellum (Kheradmand & Zee, 2011). However, in MGS tasks, there is no visual feedback when patients saccade to memorized position, which is different from reflexive saccade in SCA patients. Thus, the detailed mechanism for this phenomenon awaits further research to elucidate.

According to previous studies, reflexive saccade tests in PD patients showed mild hypometria, while in MSA patients a mild to moderate hypometria and/or hypermetria were demonstrated (Anderson et al., 2008; Anderson & MacAskill, 2013; Gorges et al., 2014; Pinkhardt et al., 2009; Termsarasab et al., 2015). Our data showed that the incidence of hypometria in MSA was prominently higher than PD, and hypermetria could only occur in MSA, which is in line with the report by Gorges et al. (Gorges et al., 2014). Hypometria in PD may be attributed to the irregular pulse in omnipause neurons of the brainstem or cortical dysfunction, whereas hypometria and hypermetria in MSA were mainly due to impairment of the cerebellum (Gorges et al., 2018; Gorges et al., 2013; R. John Leigh, 2015). So compared with PD, hypometria and hypermetria may be a unique ocular change in MSA. However, to quantitatively analyze the parameters of reflexive saccade tasks, PD and MSA showed irregular changes in accuracy and velocity, while PD showed the trend of shortening latency compared with healthy controls, which is inconsistent with Gorges et al (Gorges et al., 2016). This may be due to the cortical pathway of the reflex saccade is mainly located in the parietal lobe, which can directly send impulses to the brain stem to trigger eye movements (Sharma et al., 2011). What is more, the PD patients in this study were mainly in the early stage, so we speculate that the function of the parietal cortex or the burst neurons in brainstem may be enhanced compensatively, which still needs further research.

Inconsistent with previous studies, our study also showed that the gain of SPM tests slightly decreased in PD patients, and the decreased gain was more pronounced in MSA patients than PD and healthy controls (Anderson et al., 2008; Anderson & MacAskill, 2013; Gorges et al., 2014; Pinkhardt et al., 2008, 2009; White et al., 1983). In agreement with the literature, PD patients performed repeatedly interspersed “anticipatory saccades” and “saccadic intrusions,” while MSA showed significantly lower gain and necessitate “catch‐up saccades” (Gorges et al., 2013; Gorges et al., 2014; Pinkhardt et al., 2009). According to Pinkhardt et al., the saccade intervening in MSA may attribute to the lesions of the pontine nuclei and the cerebellar vermis and flocculus, while PD patients have considerable difficulties suppressing undesired saccade in SPM may be mainly attributed to the higher functions located in the dorsolateral prefrontal cortex as well as the striatal projections (Pinkhardt & Kassubek, 2011; Pinkhardt et al., 2009). So this differential pattern appears to allow for a differentiation of SPM pathology in MSA and PD, although the detailed pathology still needs further research. In addition, considering some participants in this study had mild cognitive dysfunction (MMSE score lower than 26), the influence of cognitive function on their ocular motor performance cannot excluded, especially in memory‐guided saccade and smooth pursuit movement (Fukushima et al., 2013; Pierrot‐Deseilligny et al., 2002).

Gaze‐evoked nystagmus, square‐wave jerks in gaze‐holding test may be clues for cerebellar deficits in MSA. Here, we found only 24.2% gaze‐evoked nystagmus and 6.1% square‐wave jerks in MSA patients, which was lower than Anderson's results, 52% and 90%, respectively (Anderson et al., 2008). We speculate that the disease duration in our study (4 years) was shorter than Anderson's study (6.6 years). Therefore, long‐term follow‐up of the eye movement is needed.

In our study, the sensitivity of multiple‐step pattern in MGS in PD was not so good as Blekher's result. We postulate that the enrolled PD patients in our study were mild to moderately affected (H&Y1 ~ 2.5 92.7%), which also had contributed to the observed differences. It is yet not certain whether these abnormal eye movement such as multiple‐step pattern or saccadic interventions in SPM will occur in other movement‐related diseases, and whether these abnormalities can well differentiate them. In addition, this study has limitations as follows: the associations between these oculomotor impairments and severities of motor dysfunction in PD or MSA are not clear, and the eye movement abnormalities of MSA‐P and MSA‐C are not compared, which need enlarge sample and further analysis.

## 5 CONCLUSION

Our study confirmed that multiple‐step pattern could also occur in MSA. PD and MSA demonstrated remarkable performance in eye movement figures in SPM and similar performance in MGS. Combined ocular tests may serve as a valuable means to assist in the differential diagnosis of PD and MSA.

## CONFLICT OF INTEREST

The authors report no conflicts of interest in this work.

## AUTHOR CONTRIBUTIONS

H.Z, G.Z, Z.W: Responsible for the conception and design of the study. H.Z, X.W, D.M, Y.J, F.L, Y.S, J.C, W.S: In charge of data collection and analysis of our study. H.Z: Manuscript drafting. G.Z and Z.W: Responsible for the critical revision of the manuscript. E.P, B.L, A.L， and L.Z: Approval of the final version of the manuscript. G.Z and Z.W: Consideration of joint senior authors.

## Supporting information

Supplementary Material

## Data Availability

The data that support the findings of this study are available from the corresponding author upon reasonable request.
