# Supplementary material for: The differential diagnostic value of a battery of oculomotor evaluation in Parkinson's Disease and Multiple System Atrophy
Source: Brain Behav. 2021 May 30;11(7):e02184. doi: 10.1002/brb3.2184 (PMC8323034; doi:10.1002/brb3.2184)
Supplement: Supplementary file 1 — Supplementary Material [file BRB3-11-e02184-s001.docx]

Supplementary materials


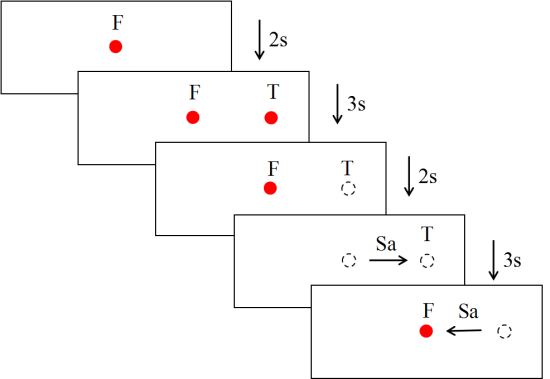


Figure 1. Schematic representation of MGS. The participant was instructed to fixate their gaze on the central spot for 2s, and then a horizontal “Target” was presented for a period of 3s at the same time. The participant was asked to keep fixating on the central spot until the target switched off, and the participant was asked to keep fixating on the central spot, which was present for 2 s. When the central spot disappeared, the participant was asked to saccade immediately toward the remembered location of the target and fixating the memorized location for 3s. When the central spot re-appeared again, the participant was instructed to fixate their gaze on the central spot for the next test.

F=Fixation, T=Target, Sa=Saccade

Figure 2 The gain of SPM was calculated separately by computer for the left and the right directions automatically. The black line is the reference line, the red line is the track of eye movement toward right, and the green line is the track of the eye movement toward left.

 Figure 3 Smooth pursuit movement of PD patients. The upper picture shows an 83-year-old female PD patient with “catch-up saccades”. The middle picture shows a 71-year-old female PD patient with “anticipatory saccades”. The last picture shows a 61-year-old male PD patient with “saccade intrusions”

Figure 4 Smooth pursuit movement of MSA patients. The upper picture shows a 47-year-old female MSA patient with “catch-up saccades”. The second picture shows a 67-year-old female MSA patient with “anticipatory saccades”, “saccade intrusions”, and “catch-up saccades”. The third picture shows a 56-year-old female MSA patient with “anticipatory saccades”. The last picture shows a 66-year-old male healthy participant with “saccade intrusions”.
